# Knockdown of Lamin B1 and the Corresponding Lamin B Receptor Leads to Changes in Heterochromatin State and Senescence Induction in Malignant Melanoma

**DOI:** 10.3390/cells11142154

**Published:** 2022-07-08

**Authors:** Lisa Lämmerhirt, Melanie Kappelmann-Fenzl, Stefan Fischer, Michaela Pommer, Tom Zimmermann, Viola Kluge, Alexander Matthies, Silke Kuphal, Anja Katrin Bosserhoff

**Affiliations:** 1 Institute of Biochemistry, Friedrich-Alexander University Erlangen-Nürnberg (FAU), Fahrstraße 17, 91054 Erlangen, Germany; lisa.laemmerhirt@fau.de (L.L.); michaela.pommer@fau.de (M.P.); tom.zimmermann@fau.de (T.Z.); viola.kluge@fau.de (V.K.); alexander.matthies@fau.de (A.M.); silke.kuphal@fau.de (S.K.); 2 Faculty of Computer Science, Deggendorf Institute of Technology, Dieter-Görlitz-Platz 1, 94469 Deggendorf, Germany; melanie.kappelmann-fenzl@th-deg.de (M.K.-F.); stefan.fischer@th-deg.de (S.F.)

**Keywords:** melanoma, nuclear lamina, chromatin state, heterochromatin foci, senescence, LMNB1, LBR

## Abstract

Modifications in nuclear structures of cells are implicated in several diseases including cancer. They result in changes in nuclear activity, structural dynamics and cell signalling. However, the role of the nuclear lamina and related proteins in malignant melanoma is still unknown. Its molecular characterisation might lead to a deeper understanding and the development of new therapy approaches. In this study, we analysed the functional effects of dysregulated nuclear lamin B1 (LMNB1) and its nuclear receptor (LBR). According to their cellular localisation and function, we revealed that these genes are crucially involved in nuclear processes like chromatin organisation. RNA sequencing and differential gene expression analysis after knockdown of LMNB1 and LBR revealed their implication in important cellular processes driving ER stress leading to senescence and changes in chromatin state, which were also experimentally validated. We determined that melanoma cells need both molecules independently to prevent senescence. Hence, downregulation of both molecules in a BRAF^V600E^ melanocytic senescence model as well as in etoposide-treated melanoma cells indicates both as potential senescence markers in melanoma. Our findings suggest that LMNB1 and LBR influence senescence and affect nuclear processes like chromatin condensation and thus are functionally relevant for melanoma progression.

## 1. Introduction

The lamina of the cell nucleus is a fibrillar structural component, which is located beneath the nuclear envelope and consists of lamins, which form lamin filaments. Moreover, the nuclear lamina is linked to the nuclear envelope through several transmembrane proteins and serves as point of anchoring for chromatin and transcription factors [1,2,3]. Lamins define the nuclear shape, participate in stress response, regulate gene expression, influence DNA replication and repair, and contribute to cell cycle progression [4,5,6,7]. The mammalian nuclear lamina is composed of type V intermediate filaments coded by three lamin genes: A-type lamin (LMNA, coding for lamin A and lamin C) and B-type lamins LMNB1 (coding for lamin B1) and LMNB2 (coding for lamin B2 and its splice variant lamin B3) [3,8,9]. Lamins undergo different posttranslational modifications, for example, farnesylation, phosphorylation, ubiquitination and methylation [10,11]. It is assumed that these proteins have different functions within the lamina structure because A-type and B-type lamins exhibit limited colocalization and A-type lamins possess a higher nuclear mobility than B-type lamins [3]. A study of Goldberg et al. showed that LMNA forms filaments as thick bundles and layers on top of the nuclear envelope which are linked to its mechanical rigidity, while B-type lamins form thin, highly organized layers that are closely associated with membranes [12]. The lamin B receptor (LBR) belongs to nuclear transmembrane proteins, interacting with chromatin at the so-called lamina-associated domains and embed B-type lamins into the nuclear lamina [3,13]. It also mediates the peripheral connection of heterochromatin to the inner nuclear membrane and plays a role in repressing transcription [14,15]. However, the attachment of LMNA/C to the nuclear lamina is not mediated by the LBR but by specific LMNA/C-binding proteins. Lamin dysregulation is linked to cancer biology, for example, the upregulation of LMNB1 [14] in primary prostate cancer (PC) as well as in breast cancer is related to poor disease-free survival [16,17]. LMNB1 is also discussed as a useful biomarker detecting hepatocellular carcinoma (HCC) in early stages [18]. Changes in the expression of lamins have been linked to various tumour entities; however, the relationship appears to be complex, and less is known about the role of LMNB1 and the LBR in melanoma. Both molecules were recently discussed in the framework of affecting the chromatin structure through rearrangement [13,15]; however, this aspect is also poorly investigated in melanoma. Therefore, in this study, we address the functional role of LMNB1 and the LBR in melanoma.

## 2. Materials and Methods

### 2.1. Cultivation of Melanocytes

Normal human epidermal melanocytes (NHEMs) were obtained from Lonza (Basel, Switzerland) and derived from human neonatal foreskin tissue of Caucasian donors. The melanocytes were grown in a melanocyte medium with PMA from Lonza (Basel, Switzerland) at 37 °C and 5% CO_2_. When the cells reached an approximately 80% confluence in a T75 cell culture flask, they were washed with phosphate-buffered saline (PBS, Sigma-Aldrich, Steinheim, Germany) and detached using a solution of 0.05% trypsin and 0.02% EDTA in PBS. After centrifugation and removal of the trypsin solution, the cells were passaged.

### 2.2. Melanoma Cell Culture

All the used cell lines were described previously [19]. The primary human melanoma cell line MEL-JUSO was cultivated in the Roswell Park Memorial Institute culture medium (RPMI) supplemented with 10% foetal bovine serum (FBS), penicillin (400 U/mL), streptomycin (50 µg/mL) and 0.2% sodium bicarbonate (all from Sigma-Aldrich, Steinheim, Germany). The cell lines derived from malignant melanoma metastases SK-MEL-28 (RRID:CVCL_0526), MEL-IM (RRID:CVCL_3980) and MEL-JU were cultivated in low-glucose Dulbecco’s Modified Eagle’s Medium (DMEM) supplemented with 10% FBS, penicillin (400 U/mL) and streptomycin (50 μg/mL) (Sigma-Aldrich). The human primary melanoma cell lines SBCL2, WM3211, WM793 and WM1366 as well as the cells derived from melanoma metastases WM1158 and WM9 were cultivated in a culture medium consisting of MCDB153 (Sigma-Aldrich) with 20% Leibowitz’s L-15 (PAA Laboratories, Pasching, Austria), 2% FBS, 1.68 mM CaCl_2_ (Sigma Aldrich), 5 µg/mL insulin (Sigma-Aldrich), penicillin (400 U/mL) and streptomycin (50 µg/mL) (Sigma-Aldrich). The melanoma cells were incubated in a humidified atmosphere containing 8% or 5% CO_2_ at 37 °C in T75 cell culture flasks (Corning Incorporated, New York, NY, USA). When the cells reached an approximately 80% confluence in a T75 cell culture flask, they were washed with phosphate-buffered saline (PBS, Sigma-Aldrich) and detached using a solution of 0.05% trypsin and 0.02% EDTA in PBS. After centrifugation and removal of the trypsin solution, the cells were either passaged or counted using a Neubauer counting chamber.

### 2.3. Lentiviral Transduction of Melanocytes

Lentiviral transduction was carried out as described previously [20]. Packaging cells (HEK293T) were transfected with a third-generation vector system. For transfections, pCMVΔR8.2, pHIT G and the plasmid DNA of interest (copGFP and B-Raf^V600E^) were mixed with DMEM (without phenol red) and, subsequently, 24 µL Lipofectamine Plus (Thermo Fisher Scientific, Waltham, MA, USA) were added to a final volume of 160 µL (mixture A). Twenty microliters of Lipofectamine LTX (Thermo Fisher Scientific) were mixed with 140 µL DMEM (without phenol red) (mixture B). After incubation for 10 min, mixtures A and B were combined, incubated for 30 min at RT and finally added to HEK293T cells, which were seeded the day before in 10 mL high-glucose DMEM into a 10 cm dish (2,000,000 cells). After incubation for 16 h (37 °C and 5% CO_2_), the cell medium was changed to the MGM-4 BulletKit medium, the melanocyte medium with PMA from Lonza (Basel, Switzerland). Twenty-four hours later, lentiviral supernatants were collected and filtered for the subsequent infection of the target cells (NHEMs at passage 7 or 8). The infected cells were incubated for 6 h, and the medium was subsequently changed to remove the virus supernatant. RNA and protein samples were obtained, and all the following experiments were performed 7 days after transduction.

### 2.4. Induction of Senescence in Melanoma Cells with Etoposide

Etoposide treatment of the melanoma cell lines MEL-JUSO and SK-MEL-28 started 24 h after approximately 300,000 cells/well were seeded in six-well plates. Etoposide (R&D System, Minneapolis, MN, USA) was dissolved in DMSO to achieve a stock solution of 50 mM, which was then diluted in a culture medium to a final concentration of 100 µM and applied to the cells. The controls were treated with a similar amount of DMSO to exclude effects of the solvent. After an incubation period of 48 h, the cells were detached as described in 2.2. “Melanoma Cell Culture” and collected for further processing.

### 2.5. siRNA Transfection

The melanoma cell lines MEL-JUSO or SK-MEL-28 were transfected with a siRNA pool for LMNB1 or the LBR (siTools Biotech GmbH, Planegg, Germany) and a siCtrl, respectively, using the Lipofectamine RNAiMAX reagent (Life Technologies, Darmstadt, Germany). SiRNA pools consist of multiple siRNAs resulting in efficient target gene knockdown with minimal off-target effects [21]. For 72 h transfection, 150,000 cells were seeded into wells of a six-well plate. For long-term transfection, approx. 25,000 cells were used. The cells were detached via trypsin, stopped with the respective medium, counted, reseeded, and transfected for 96 h every week for at least three months.

### 2.6. Immunofluorescence Staining

For the verification of the nuclear protein PML, some immunofluorescence analyses were performed. Therefore, the melanoma cells were treated with siRNA for 72 h, then harvested, counted, and up to 25,000 or 30,000 cells were sawn for each siRNA treatment onto round 18 mm cover slides (Carl-Roth, Karlsruhe, Germany) in 12-well culture plates (Corning Incorporated, Corning, USA). Before staining, the cells were incubated for 24 h at 37 °C. Then, the cover slides were washed two times with PBS. After 5 min of fixation and permeabilization with ice-cold methanol, the cells on the cover slides were blocked with 10% bovine serum albumin (BSA) in PBS. The cover slides were incubated overnight with the primary anti-PML antibody (1:200, sc-966, Santa Cruz Biotech, Heidelberg, Germany) at 4 °C or for 1 h at room temperature with the primary antibody LMNB1 (1:1000, ab133741) or the LBR (1:500, ab32535) (Abcam, Cambridge, UK) in 1.5% BSA/PBS. On the following day, the slides were incubated for 1 h with the secondary Cy3 antibody (1:500, Biozol, Eching, Germany). Subsequently, the slides were incubated in a DAPI solution (Merck KGaA, Darmstadt, Germany) in 1% BSA/PBS for 30 min. After every incubation step, the slides were washed several times with PBS. Aqua-Poly/Mount (US Headquarters Polysciences, Warrington, PA, USA) was used as the mounting medium. Immunofluorescence staining was analysed with an IX83 microscope with the Olympus CellSens Dimension software (version 2.3, Olympus, Hamburg, Germany).

### 2.7. Senescence-Associated β-Galactosidase Staining

For senescence-associated β-galactosidase staining (SA β-Gal) [22], a staining kit from Cell Signaling (#9860S, Frankfurt, Germany) was used according to the manufacturer’s protocol [23]. MEL-JUSO and SK-MEL-28 long-term-treated with siLMNB1 and siLBR, as well as the 72 h-transfected ones, were seeded in six-well culture plates and incubated in the staining solution for 5.5–8 h. Six microscope images were taken from each treatment at 10-fold magnification. All visible cells and SA-β-Gal-positive cells were counted, and the normalized percentage of SA-β-Gal-positive cells to the total cells was reported. For the ratio of the percentage of β-Gal-positive cells between siLMNB1 and siLBR after 72 h transfection and LTT, the percentage of β-Gal-positive cells of the respective siCtrl was first subtracted before the ratio was calculated. Therefore, the respective siCtrl is already included in the ratio.

### 2.8. Western Blot Analysis

Cell pellets were lysed in 60 µL RIPA buffer (Roche, Manheim, Germany) for 15 min at 4 °C. During centrifugation (13,000× *g* rpm, 10 min, 4 °C), cell fragments were removed, and the supernatant was collected. For protein amount quantification, the Pierce^TM^ BCA Protein Assay Kit (Thermo Fisher Scientific, Waltham, MA, USA) was used. Then, 30 µg of total RIPA lysates were loaded on polyacrylamide gels. After protein separation, the gel was blotted on a PVDF membrane (GE Healthcare, Chicago, IL, USA). Each membrane was blocked with 5% milk powder/TBS-T. Subsequently, the membrane was incubated with the primary anti-LMNB1 (1:1000, ab133741), anti-LBR (1:500, ab32535, Abcam, Berlin, Germany) and anti-H3 (1:1000, #4499, Cell Signaling, MA, USA) in 5% milk powder/TBS-T. As a loading control, anti-β-actin (1:5000, #A5441, Sigma-Aldrich, Steinheim, Germany) in TBS-T was used. After washing three times for 10 min with TBS-T, the membrane was incubated with a horseradish peroxidase-coupled secondary antibody (1:2000, anti-rabbit HRP or anti-mouse HRP, Cell Signaling Technology) for 1 h. Immunoreactions were visualized by ECL staining (Bio-Rad, Feldkirchen, Germany). For the expression analysis of LMNB1 and the LBR in melanoma, the relative protein level was first normalized to the housekeeper β-actin and then normalized to the early primary melanoma cell line SBCL2 because both genes are rarely expressed in NHEMs.

### 2.9. RNA Isolation and Reverse Transcription from Mammalian Cells

RNA extraction from the cell pellets was performed with E.Z.N.A. Total RNA Kit I (Omega Bio-Tek, Inc., Norcross, GA, USA) according to the manufacturer’s instructions. RNA concentrations were measured with a Nanodrop 2000. The transcription of mRNA into cDNA (complementary DNA) was accomplished with the SuperScript^®^ II Reverse Transcriptase (Thermo Fisher Scientific, Waltham, MA, USA). The reaction components for the reverse transcription were 5× First-Strand Buffer (4 µL), DTT (0,1 M, 2 µL), dNTPs (10 µM, 1 µL), dN6 Primer (1 µL) and 500 ng of the previously obtained total cellular RNA. The volume was replenished up to 19 µL with RNAse-free water. At first, all the samples were incubated for 5 min at 70 °C to denature the RNA. After a short cooling time, 1 µL of the Superscript II Reverse Transcriptase was added to each sample. Then, the samples were incubated for 1 h at 37 °C, followed by heat inactivation at 70 °C for 10 min. To remove the already existing RNA, digestion via RNase for 20–30 min at 37 °C was performed. The received cDNA was stored at −20 °C before use.

### 2.10. Quantitative RT-PCR with mRNA

Quantitative real-time PCR (qRT-PCR) analysis of gene expression was performed on a LightCycler 480 system with specific sets of primers as described previously [24]. The primer sequences were as follows: hLMNB1_for185: TATGAGTACAAGCTGGCGCA, hLMNB1_rev370: TCTCATGCGGCTTTCCATCA, hLBR_for194: GCACCTCCCAGCTTTACACT, hLBR_rev422: TCCTTAATGTCGGCCTGGTG, hLIFR_2254for: TGAGGGTTTTAGAATCAGGTCGTT, hLIFR_2474rev: CACTGCCACTGGGATGAGAAT, hMMP16_for346: CGTCGAAAGCGATATGCATTG, hMMP16_rev458: CACACATCAAAGGCACGGC.

### 2.11. Chromatin Accessibility Assay

The chromatin accessibility assay was performed with the Chromatin Accessibility Assay Kit of EpiQuik^TM^ (Epigentek Group Inc., Farmingdale, NY). The melanoma cell lines MEL-JUSO were treated with siLMNB1, siLBR and siCtrl, respectively, for 72 h or long-term transfection. After the incubation, 1 × 10^6^ cells per treatment were harvested and washed with 1 mL PBS. Centrifugation for 5 min (1000× *g*) was followed by rejection of the supernatant and resuspension in 400 µL 1× lysis buffer. Then, 200 µL of the cell suspension were transferred to a new 1.5 mL vial as a sample with the Nuclease Mix (NSE). The remaining 200 µL of the cell suspension were transferred to another 1.5 mL vial as the No-NSE control. The cell suspension was incubated for 10 min on ice, vortexed for 10 s and centrifuged for 5 min at 5000× *g*. Subsequently, the supernatant was carefully removed, and the chromatin pellet was washed once with 1 mL of 1× wash buffer by resuspending the pellet and centrifuging at 3000× *g* for 5 min (4 °C). The wash step was repeated with 0.5 mL of the 1× wash buffer. The supernatant was discarded. The NSE reaction mixtures were separately prepared as follows: sample: 48 µL NDB, 2 µL NSE; No-NSE control: 50 µL NDB. After mixing, 50 µL of the respective solution were added to the chromatin sample and the No-NSE control. The tubes were incubated at 37 °C for 4 min after resuspension. Then, 10 µL of the reaction stop solution were added to each tube and incubated for 10 min at 37 °C. Subsequently, 2 µL of Proteinase K were added to each tube and incubated for 15 min at 60 °C. After DNA clean-up according to the user manual, the purified DNA was analysed via real-time qPCR using primers for different repeats. Fold enrichment (FE) was calculated as the ratio of amplification efficiency (Ct) of the digested DNA sample over that of the nondigested sample. Large Ct shifts between digested and undigested samples rendering high FE% indicated that the target region was in open chromatin, while minimal Ct shifts, resulting in low FE%, indicated that the target region was in closed chromatin. For evaluation, log10 %FE was shown in the respective graphs. The chromatin state is defined by a cut-off range. If log10 %FE < 2.6, the chromatin is closed, if log10 %FE > 3.2, the chromatin is open, and everything in between can be seen as a respective tendency.

### 2.12. Cell Cycle Analysis with Propidium Iodide Flow Cytomentry

The cell cycle of the melanoma cell lines MEL-JUSO and SK-MEL-28 treated with siLMNB1 and siLBR (72 h) was analysed by flow cytometry. The cells were transfected for 72 h with siLMNB1 or siLBR and siCtrl, respectively. For each treatment, 200,000 cells were sown, harvested and fixed in 70% ice-cold methanol for at least 1 h. Then, the cells were washed twice with 0.2% BSA/PBS. After centrifugation (4000 rpm, 4 min), the cells were resuspended in 482.5 µL of 0.2% BSA/PBS, followed by addition of 10 µg/µL Rnase to each tube and incubation for 20 min at 37 °C. Just before the measurement, 12.5 µL of propidium iodide (1 mg/mL PromoCell, Heidelberg, Germany) were added to each tube, gently mixed, and incubated for 30 min. Propidium iodide is a fluorescent dye which intercalates into double-stranded nucleic acid, and therefore the DNA content could be measured. Analysis was carried out using a BD LSRFortessa^™^ X20 flow cytometer, and flow cytometry data were analysed using the BD FACSDIVA software (version 8.0, BD Bioscience, San Jose, CA, USA).

### 2.13. RNA-Seq Library Preparation, Data Preprocessing and Analysis

The total RNA samples were isolated using Total RNA kit I (Omega Bio-Tek, Inc., Norcross, GA, USA) according to the manufacturer’s instructions. All the RNA samples were examined for integrity and purity with a TapeStation 4200 (Agilent). Library preparation was performed with three biological replicates using the TruSeq^®^ Stranded Total RNA Library Prep Human/Mouse/Rat Kit according to the manufacturer’s instructions (Illumina Inc., San Diego, CA, USA). The resulting libraries were checked for size (200–500 bp) using the TapeStation 4200 (Agilent) using the High-Sensitivity DNA Kit (Agilent), for concentration—using a Qubit 4 Fluorometer (Thermo Fisher). Sequencing was performed according to the paired-end RNA sequencing protocols from Illumina on a HiSeq4000 with a paired-end module (Illumina, Inc., San Diego, CA, USA). The samples were sequenced from each side of a fragment approximately 75 bp long, with an average number of 20 million reads per sample. After quality check using FastQC [25] (v0.11.9, accessed on 30 March 2021), paired-end reads were aligned to the human reference genome using the STAR alignment software (v2.7.9a) [26]. After mapping, only reads that mapped to a single unique location were considered for further analysis. The mapped reads were then used to generate a count table using the featureCounts software (v2.0.1) [27]. The raw reads were filtered, normalized and visualized by using R (v4.0.5, The R Foundation for Statistical Computing) [28]. The DESeq2 package (v1.28.1) [29] was used for logarithmic transformation of the data and for data exploration. Differential expression analysis was performed using the DESeq2 standard approach. Adjusted *p*-values are calculated using the Benjamini–Hochberg method within DESeq2. Gene annotations were added to the result files using Ensemble data. Differentially expressed genes with an adjusted *p*-value < 0.1 were regarded as statistically significant. Functional data analysis was performed using the Gene Set Enrichment Analysis Tool (GSEA; v4.2.3) [30,31] and the Search Tool for the Retrieval of Interacting Genes/Proteins (STRING; v11.5) [32]. GSEA analysis was performed using the unfiltered list of normalised counts. The analysis using C2 Canonical pathways, C5 GO biological processes, molecular functions and cellular components as well as hallmarks using gene sets from MsigDB (v7.5.1) [31,33] was performed with classical weighting, the Signal2Noise metric and 1000 permutations of gene sets. Subsequent protein–protein interaction (PPI) networks of the products of the differentially expressed genes were produced by Cytoscape (v3.9.1) [34] and the STRING application (v1.7.0) [32,35]. Further enrichment analysis of the PPI was performed with the same application. Enrichment results were considered significant with FDR < 0.25.

### 2.14. Statistical Analysis

Statistical analysis was performed using GraphPad Prism 9 software package (version 9.3.1, GraphPad Software Inc., San Diego, CA, USA). The results were shown as the means ± standard error of the mean (SEM). Comparisons between the groups were conducted using Student’s unpaired *t*-test, one-way ANOVA or two-way-ANOVA, respectively. Unless otherwise indicated, the number of independent experiments was *n* = 3. The ΔCP value *p* ≤ 0.05 was considered statistically significant (ns = not significant).

## 3. Results

### 3.1. LMNB1 Expression in Melanoma and its Relevance for Chromatin Structure

**Figure 1:**
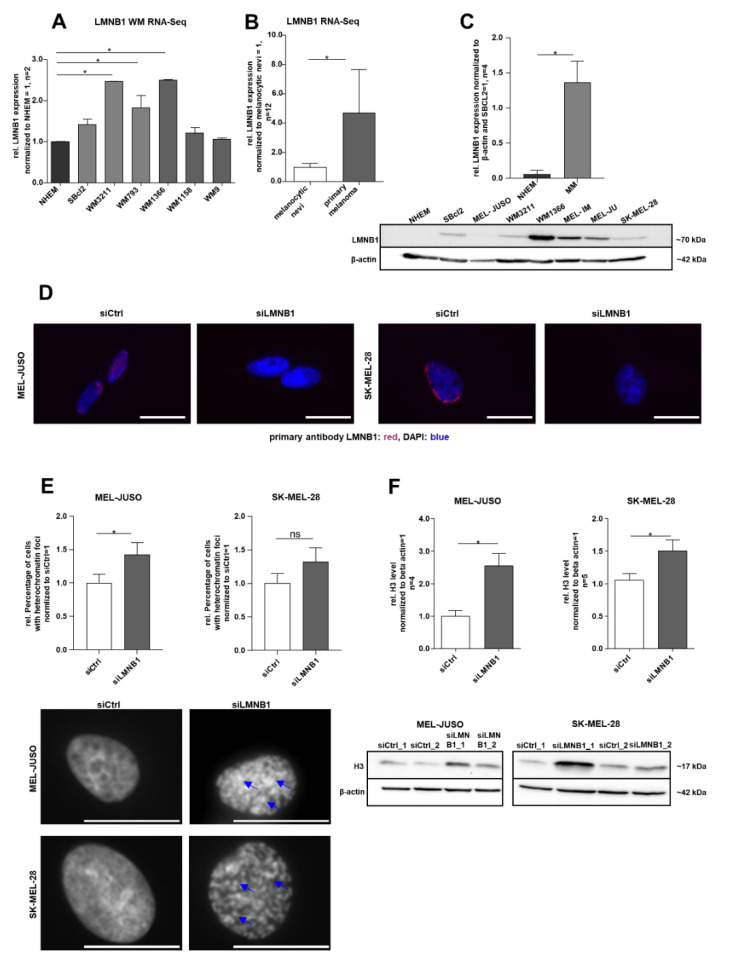
(**A**) RNA sequencing analysis of LMNB1 expression in different Wistar melanoma (WM) cell lines compared to NHEMs (one-way ANOVA and subsequent Tukey’s multiple comparison test). (**B**) RNA sequencing analysis of LMNB1 expression in the primary melanoma compared to melanocytic nevi (one-way ANOVA and subsequent Tukey’s multiple comparison test). (**C**) Western blot analysis of LMNB1 protein expression in NHEMs and various melanoma cell lines (Student’s *t*-test). (**D**) Representative images of immunofluorescence LMNB1 staining in MEL-JUSO and SK-MEL-28 transfected with siLMNB1 and siCtrl for 72 h. Scale bars equal 20 µm. (**E**) DAPI staining of MEL-JUSO and SK-MEL-28 cells transfected with siLMNB1 and siCtrl for 72 h. Visible heterochromatin foci marked with blue arrows. Statistical analysis was performed using Student’s *t*-test. Scale bars equal 20 µm. (**F**) Western blot analysis of the H3 level following 72 h treatment with siLMNB1 and siCtrl in MEL-JUSO and SK-MEL-28 using β-actin as a housekeeper (Student’s *t*-test). Bars represent the means ± SEM (* = *p* ≤ 0.05, ns = not significant).

To define the molecular function of LMNB1 in melanoma, we first evaluated its gene expression in the RNA sequencing (RNA-Seq) data of different melanoma cell lines compared to NHEMs (PRJNA839865) and in the primary melanoma compared to melanocytic nevi [36] (GSE112509). Differential gene expression analysis revealed a significant (adj. *p* < 0.1) upregulation of LMNB1 in melanoma cell lines and tissues compared to the respective control, NHEMs or nevi (Figure 1A,B). The observed upregulation of LMNB1 on the RNA level could be experimentally validated on the protein level by Western blot analysis (Figure 1C). Further, immunofluorescence staining confirmed the presence and localisation of LMNB1 in the nucleus (Figure 1D). Since LMNB1 is a nuclear structure protein involved in chromatin organisation and DNA replication, we examined the influence of LMNB1 on heterochromatin organisation using LMNB1-specific siRNA (siLMNB1) transfection experiments. Subsequently, we performed DAPI staining for detection of chromatin condensation in MEL-JUSO (derived from the primary melanoma) and SK-MEL-28 (derived from melanoma metastases) transfected for 72 h with siLMNB1 or the respective control siRNA (Figure 1E). An increased number of nuclei with condensed chromatin, appearing as heterochromatin foci, was found in LMNB1 knockdown cells compared to the control transfected cells (siCtrl). The effect of LMNB1 knockdown on chromatin was supported by a significantly increased histone 3 (H3) level in MEL-JUSO and SK-MEL-28 compared to control cells (Figure 1F). Conclusively, the heterochromatin foci formation and histone level revealed significant changes and are indicative for further investigations.

### 3.2. LMNB1 Knockdown Leads to Senescence Induction

**Figure 2:**
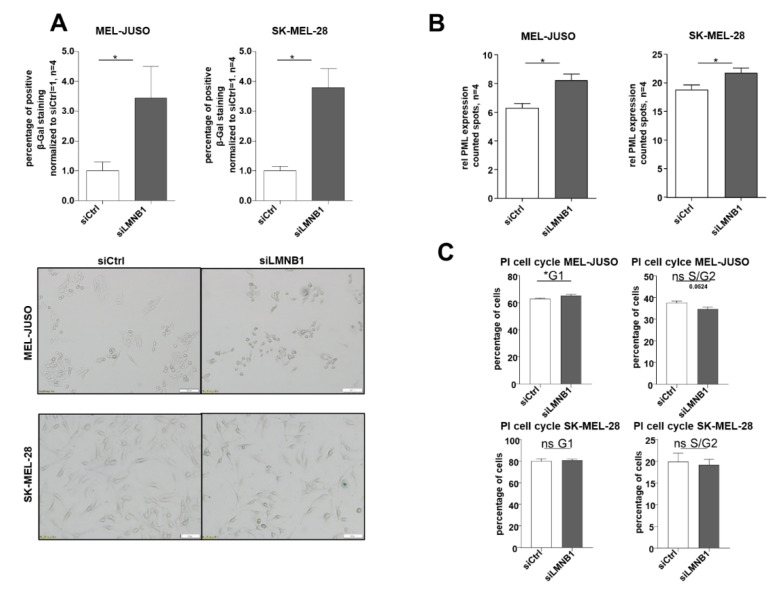
(**A**) SA β-galactosidase staining of MEL-JUSO and SK-MEL-28 transfected with siLMNB1 (72 h) as senescence detection (Student’s *t*-test). (**B**) Immunofluorescence staining of the promyelocytic leukemia protein (PML) expression in MEL-JUSO and SK-MEL-28 transfected with siLMNB1 and siCtrl for 72 h as senescence detection (Student’s *t*-test). (**C**) Flow cytometry with the fluorescent dye propidium iodide for cell cycle staining. MEL-JUSO and SK-MEL-28 treated with siLMNB1 and siCtrl for 72 h (Student’s *t*-test). Bars represent the means ± SEM (* = *p* ≤ 0.05, ns = not significant).

Due to the observed strong expression of LMNB1 in melanoma cells, we further aimed to identify its functional relevance. The increased amount of heterochromatin foci in LMNB1 knockdown cells could indicate the need for LMNB1 in melanoma cells to prevent cellular ageing or senescence. To confirm this, we investigated the functional influence of LMNB1 knockdown in MEL-JUSO and SK-MEL-28 cells using established senescence assays [37,38] and revealed an increase in senescent cells as determined by SA β-Gal staining (Figure 2A). Furthermore, immunofluorescence staining of the promyelocytic leukaemia protein (PML) expression after LMNB1 knockdown indicated a significant induction of the PML (Figure 2B). The PML serves as a molecular marker for DNA damage in relation to senescence. A significant cell cycle arrest after LMNB1 knockdown, which was assumed according to the previous results, was confirmed for MEL-JUSO (Figure 2C, for the percentage of cells see Figure S1A). The effect on senescence induction was low. We hypothesize that the small effect on senescence induction after 72 h transfection with siLMNB1 or siLBR was due to the fact that other molecules like emerin (EMD), another nuclear structure protein, might compensate the function of LMNB1 or the LBR and is redundantly induced (Figure S2A,B). Although siLMNB1 has only a small effect on the cell cycle arrest, in summary, our results demonstrate a significant increase in senescent cells by downregulation of LMNB1 in malignant melanoma cells.

### 3.3. Modulating Chromatin State and Increasing Senescence by Long-Term Knockdown of LMNB1

**Figure 3:**
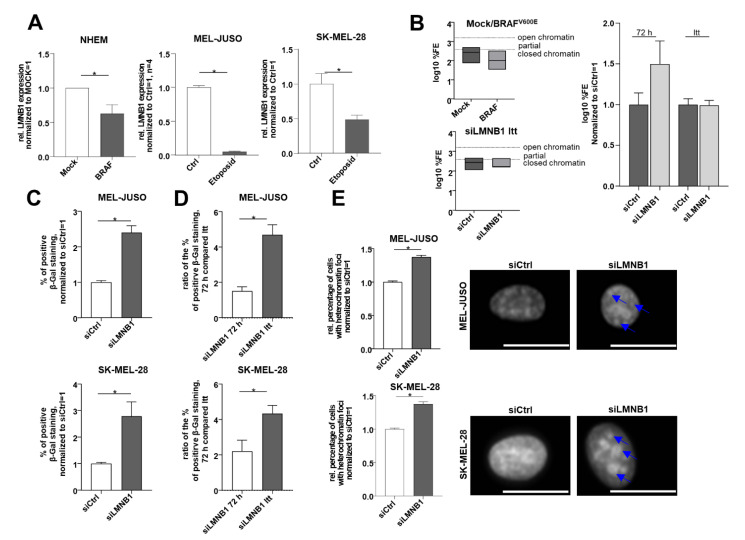
(**A**) LMNB1 expression analysed by qRT-PCR in the established senescence model using mock- and BRAF^V600E^ lentiviral-transduced NHEMs, as well as MEL-JUSO and SK-MEL-28 cells treated with the cytostatic drug etoposide compared to the control (Student’s *t*-test). (**B**) Epiquik Chromatin Accessibility Assay to verify the chromatin status in NHEM, lentiviral-transduced with Mock as control and BRAF^V600E^ (established senescence model) and siLMNB1-long-term-treated melanoma cells (MEL-JUSO) compared to siCtrl. Normalized log10 %FE for siCtrl 72 h and siCtrl LTT was used to exclude an effect of the transfection process by siCtrl over a longer period. (**C**) SA β-Gal staining of MEL-JUSO and SK-MEL-28 long-term-transfected with siLMNB1 and siCtrl as senescence detection (Student’s *t*-test). (**D**) Ratio in % of positive S β-Gal staining of MEL-JUSO and SK-MEL-28 for transfection with siLMNB1 72 h in comparison to long-term transfection. The percentage of beta-gal-positive stained melanoma cells of the respective siCtrl was included in the ratio between siLMNB1 72 h and siLMNB1 LTT (Student’s *t*-test). (**E**) The melanoma cell lines MEL-JUSO and SK-MEL-28 were long-term-transfected for three months with siLMNB1 and siCtrl, respectively. Chromatin condensation was analyzed using DAPI staining and subsequently quantified. Enlarged images show representative chromatin foci marked with blue arrows. Scale bars equal 20 µm. Bars represent the means ± SEM (* = *p* ≤ 0.05).

Next, the expression level of LMNB1 in the already senescent cells was analysed by using the well-established BRAF^V600E^-expressing melanocytic senescence model of Michaloglou et al. [39]. This model had already been used by our research group for senescence studies [23]. Interestingly, we revealed that LMNB1 is downregulated in BRAF^V600E^- compared to mock-transduced NHEMs (Figure 3A). For a proof of concept, we induced senescence in melanoma cells with the cytostatic drug etoposide, which was described previously [38]. LMNB1 was downregulated in MEL-JUSO and SK-MEL-28 cells treated with etoposide for 48 h (Figure 3A). Moreover, we determined the chromatin state in the BRAF^V600E^ senescence model. As expected, BRAF^V600E^-transduced senescent cells showed a more closed chromatin state compared to Mock-transduced cells (Figure 3B). In melanoma cells, we performed these experiments using long-term-transfection (LTT) with siLMNB1 for several weeks, at least three months. During this experiment, the chromatin state of MEL-JUSO was periodically analysed. Interestingly, we could detect a more condensed chromatin state for siLMNB1-long-term-transfected melanoma cells compared to 72 h siLMNB1-transfected MEL-JUSO (Figure 3B). Thus, it could be assumed that changed chromatin structure is in strong association to senescence after LTT. To confirm this, we performed SA β-Gal staining after siLMNB1 LTT and observed a significantly increased number of senescent cells compared to siCtrl. (Figure 3C). The ratio of the percentage of SA-β-Gal-positive cells for siLMNB1-long-term-transfected cells is also significantly increased compared to the cells transfected for 72 h (Figure 3D). These results are supported by the detection of an increased amount of heterochromatin foci in LTT melanoma cells, confirming the functional effect of LMNB1 knockdown leading to senescence (Figure 3E). Conclusively, we were able to modulate the chromatin state by long-term transfection resulting in an induction of senescence.

### 3.4. Predicted Role of LMNB1 in Melanoma Based on Transcriptome Analyses

**Figure 4:**
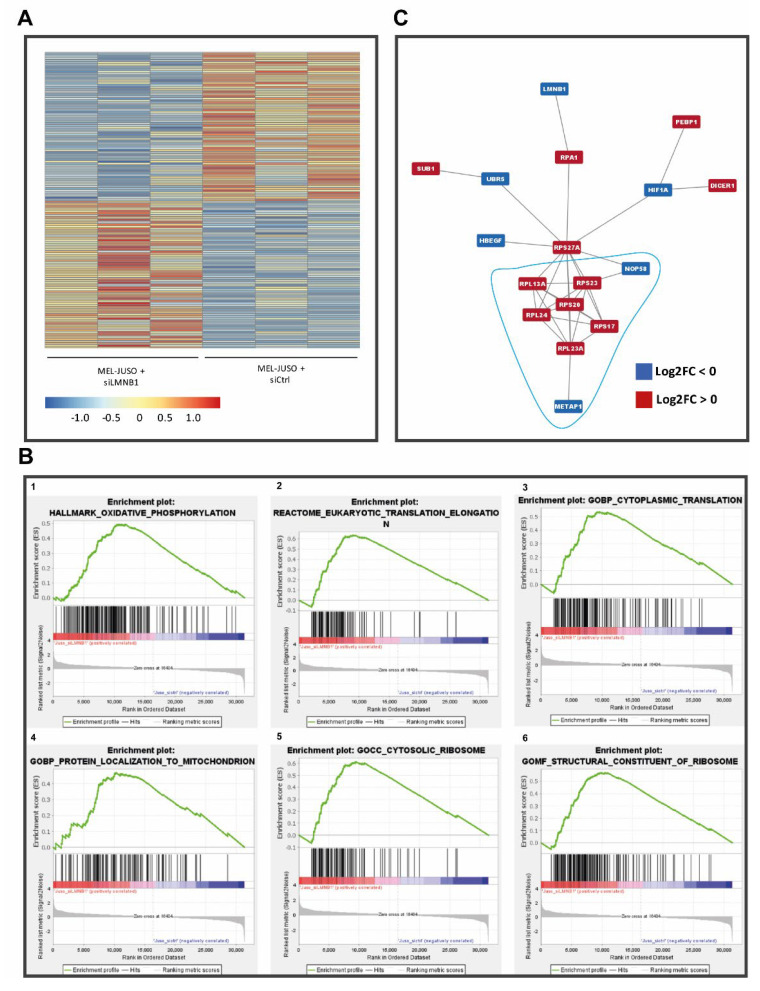
(**A**) Heatmap of differentially expressed (adj. *p* < 0.1) genes upon siLMNB1. (**B**) Enrichment plots (FDR < 25%) illustrating the profile of the running enrichment score (green) and positions of the enriched gene sets and the rank-ordered list of genes differentially expressed in MM cells treated with siLMNB1 and siCtrl, respectively, identified by GSEA. Genes upregulated in siLMNB1 MM cells are shown on the left side of the graph in red, downregulated ones—on the right side in blue. (**C**) Biggest network of protein–protein interactions (PPIs) of possibly expressed gene products from the differentially expressed genes (red: log2FC > 0; blue: log2FC < 0). Enrichment analysis showed a putative relevance of these protein networks in translation and ribosome formation, partially under stress conditions.

To further determine the molecular role of LMNB1 in melanoma and its involvement in relevant biological processes in cancer, RNA-Seq of siLMNB1-transfected primary melanoma cell lines (MEL-JUSO) was performed (PRJNA841450). Before RNA-Seq library preparation, the transfection efficiency was confirmed via qRT-PCR (Figure S3B). The data obtained from the RNA sequencing approach were pre-processed, quality-checked (FastQC v0.11.9) [25] and mapped to the human genome (GRCh38.p5, release 24) (STAR v2.7.9a) [26]. Next, a count table was generated via featureCounts (v2.0.1) [27] and used for differential gene expression analysis by the DeSeq2 package [29] in R (v4.0.5, The R Foundation for Statistical Computing) [28]. Differential gene expression analysis resulted in 66 significantly upregulated and 66 significantly downregulated genes in siLMNB1-transfected melanoma cells compared to siCtrl (adj. *p* < 0.1; differentially expressed genes (DEGs) in Table S1). The resulting target gene expression of MMP16 and LIFR was also validated with qRT-PCR in siLMNB1-transfected MEL-JUSO (Table S2). Next, we aimed to assign biological meaning to the identified differentially expressed genes of siLMNB1- versus siCtrl-transfected cells. Thus, we performed gene set enrichment analysis (GSEA, v4.2.3) [30,31] with the normalised count table obtained from the RNA-Seq data to identify the gene sets modulated by LMNB1 (Table S2) and focused on GO term subdomains (biological processes, molecular functions and cellular components), canonical pathways and hallmarks from the Molecular Signature Data Base (MSigDB; v7.5.1) [31,40] (Figure 4B). These analyses resulted in the enrichment of 64 gene sets, with 60 gene sets enriched in siLMNB1-, four-in siCtrl-transfected cells. The identified gene sets enriched in LMNB1 knockdown cells were mainly related to rRNA processing, translation, ribosome activity and protein targeting, especially to mitochondria, as mitochondrial function and translation (31 out of 60 gene sets) (Figure 4B). Additionally, gene sets including MYC targets and genes involved in DNA repair showed an enrichment in siLMNB1 cells (Table S2). In contrast, only four gene sets with no clear functional relevance to the cellular phenotype were enriched in the control-transfected cells (Table S2). Further, we investigated possible relevant protein–protein interactions (PPIs) of gene products of the differentially expressed genes (Figure 4C). The 132 genes, which are significantly regulated upon siLMNB1 versus siCtrl, resulted in only 56 physical interactions found by the STRING application (v1.7.0) [32] for Cytoscape (v3.9.1) [34]. The detected PPIs only showed six networks of protein–protein interactions. Enrichment with STRING against all the identified genes of the used RNA-Seq data revealed that PPI clusters with nine, four, three and two members were enriched for integrin and ECM interaction, complex I of the OXPHOS chain, nucleotide metabolism and cytochrome c, respectively. The biggest network of PPIs with 17 members also contains LMNB1 and seems to be relevant for translation and ribosome formation, partially connected to stress response with connections to DNA repair mechanism and cell cycle arrest.

### 3.5. Expression of the Corresponding Lamin B Receptor and its Influence on the Chromatin Structure

**Figure 5:**
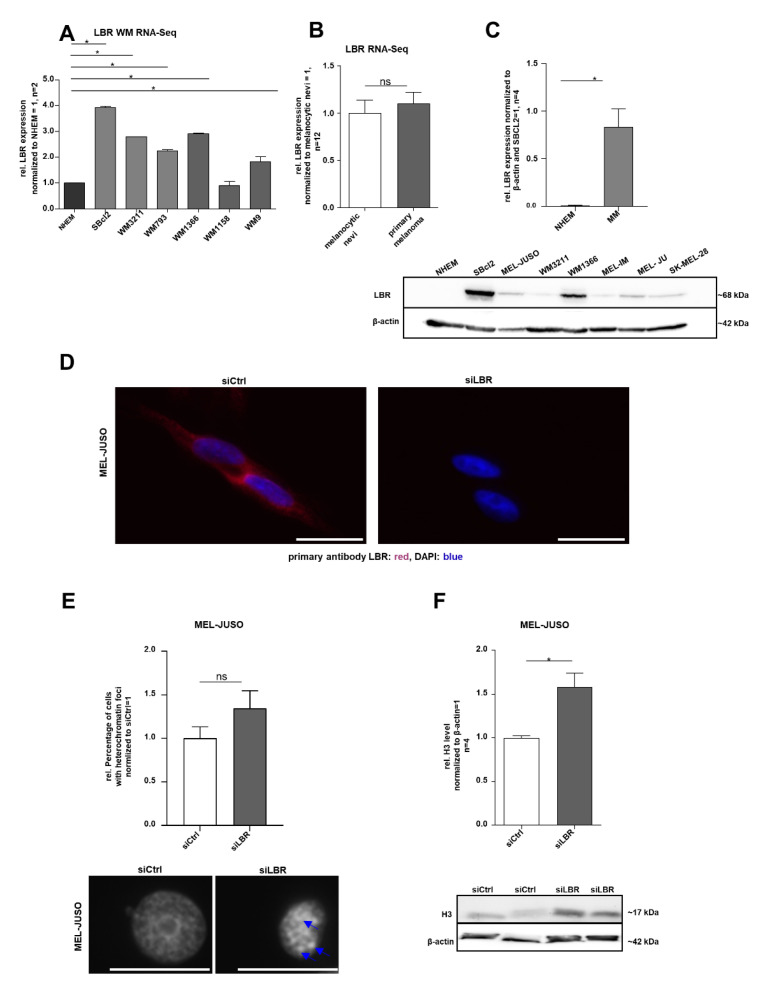
(**A**) RNA sequencing analysis of LBR expression in different WM cell lines compared to NHEMs (one-way ANOVA and subsequent Tukey’s multiple comparison test). (**B**) RNA sequencing analysis of LBR expression in the primary melanoma compared to melanocytic nevi (one-way ANOVA and subsequent Tukey’s multiple comparison test). (**C**) Western blot analysis of LBR protein expression in NHEMs and various melanoma cell lines (Student’s *t*-test). (**D**) Immunofluorescence staining of LBR expression in MEL-JUSO transfected 72 h with siLBR and siCtrl, respectively. Representative examples of LBR expression (red) in the cell nucleus stained with DAPI (blue). Scale bars equal 20 µm. (**E**) The melanoma cell line MEL-JUSO was transfected for 72 h with siLBR and siCtrl, respectively. Chromatin condensation (DAPI) was analysed and quantified (Student’s *t*-test). Enlarged images show representative chromatin foci marked with blue arrows. Scale bars equal 20 µm. (**F**) Western blot analysis of the H3 level following 72 h treatment of siLBR and siCtrl, respectively, in MEL-JUSO using β-actin as a housekeeper (Student’s *t*-test). Bars represent the means ± SEM (* = *p* ≤ 0.05, ns = not significant).

As we could demonstrate the deregulation of LMNB1 in MM, its influence on gene expression and, thus, its relevance in cancer-associated biological processes, we aimed to further investigate if these effects are also modulated by the lamin B receptor (LBR). For defining the molecular function of the LBR in melanoma, we first evaluated the gene expression in the RNA-Seq data of different melanoma cell lines compared to NHEMs (PRJNA839865) and in the primary melanoma compared to melanocytic nevi [36] (GSE112509). A differential gene expression analysis of both datasets revealed a significant (adj. *p* < 0.1) upregulation of the LBR in melanoma cell lines and tissues (Figure 5A,B). These results could be confirmed on the protein level as well as the mRNA level by Western blot (Figure 5C) and qRT-PCR analysis (Figure S3A). Immunofluorescence staining revealed the localisation of the LBR in the cell nucleus and the nuclear membrane (Figure 5D). Since the LBR is a transmembrane nuclear structure protein involved in chromatin organisation and DNA replication, similar to LMNB1, we aimed to determine the influence of siLBR transfection on heterochromatin organisation. Thus, we performed nuclear staining with DAPI for chromatin condensation detection in MEL-JUSO transfected for 72 h with siLBR (Figure 5E). The results showed a tendency to having more nuclei with condensed chromatin appearance as heterochromatin foci in LBR-knockdown cells compared to control-transfected cells (siCtrl). The significantly increased H3 protein level in MEL-JUSO treated with siLBR also underlines the changes in heterochromatin state (Figure 5F). In conclusion, for the LBR like LMNB1, heterochromatin foci formation and histone level support the impact of the LBR. Therefore, the results are indicative for further investigations.

### 3.6. Functional Role of the LBR under Short-Term and Long-Term Knockdown in Melanoma

**Figure 6:**
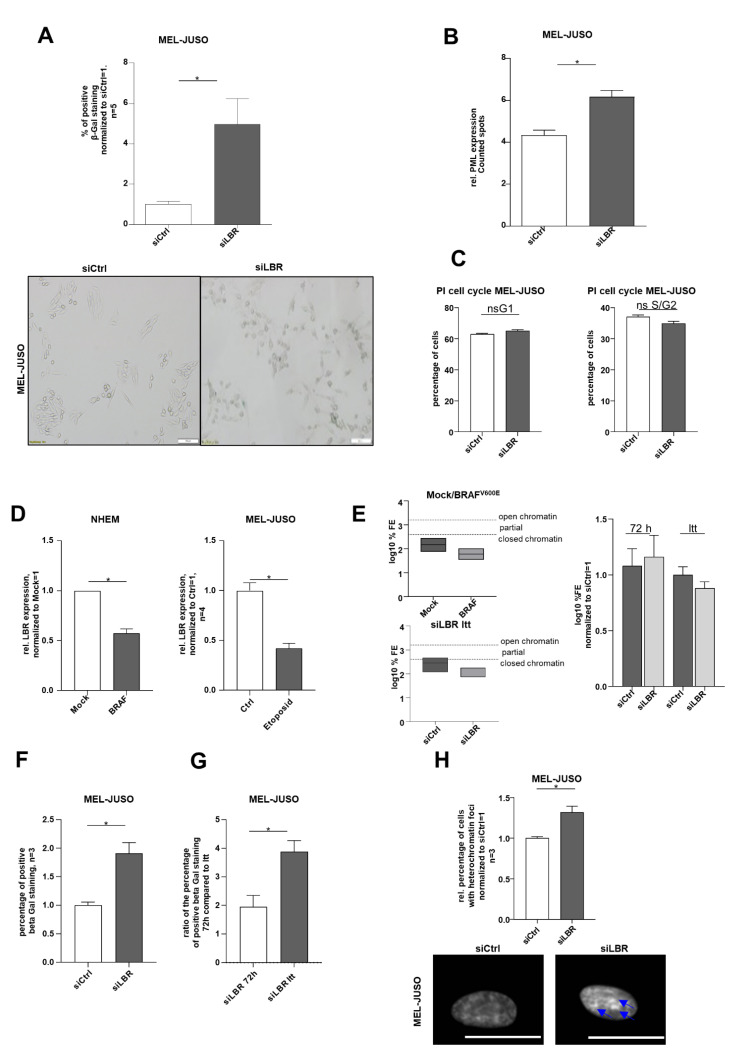
(**A**) Beta-Gal staining of MEL-JUSO transfected with siLBR and siCtrl for 72 h as senescence detection (Student’s *t*-test). (**B**) Immunofluorescence staining of the promyelocytic leukaemia protein (PML) expression in MEL-JUSO treated with siLBR and siCtrl for 72 h as senescence detection (Student’s *t*-test). (**C**) Flow cytometry with the fluorescent dye propidium iodide for cell cycle staining. MEL-JUSO treated with siLBR an siCtrl for 72 h (Student’s *t*-test). (**D**) LBR expression analyzed by qRT-PCR in the established senescence model using mock- and BRAF^V600E^ lentiviral-transduced NHEMs, as well as MEL-JUSO cells treated with the cytostatic drug etoposide compared to the control (Student’s *t*-test). (**E**) Epiquik Chromatin Accessibility Assay to verify the chromatin status in NHEMs treated with mock and Braf^V600E^ (established senescence model) and in siLBR long-term-treated MEL-JUSO compared to siCtrl. Normalized log10 %FE for siCtrl 72 h and siCtrl LTT, to exclude an effect of the transfection process by siCtrl over a longer period. (**F**) Beta-Gal staining of MEL-JUSO long-term-transfected with siLBR as senescence detection (Student’s *t*-test). (**G**) Showing the ratio of the positive β-Gal staining of MEL-JUSO for transfection with siLBR 72 h in comparison to long-term transfection. The percentage of beta-gal-positive stained melanoma cells of the respective siCtrl was included in the ratio between siLBR 72 h and siLBR LTT (Student’s *t*-test). (**H**) The melanoma cell line MEL-JUSO was long-term-transfected for three months with siLBR and siCtrl, respectively. Chromatin condensation (DAPI) was analysed and quantified (Student’s *t*-test). Enlarged images show representative chromatin foci and visible SAHF marked with blue arrows. Scale bars equal 20 µm. Bars represent the means ± SEM (* = *p* ≤ 0.05).

Due to the newly detected upregulation of the LBR in melanoma cells, we also wanted to identify the functional relevance of this change in gene expression. The increased amount of heterochromatin foci in LBR-knockdown cells indicates that the LBR might have similar functional effects as LMNB1 in melanoma. Therefore, for functional characterisation, the same assays for senescence were performed in LBR-knockdown MEL-JUSO cells. The knockdown of LBR by siLBR transfection (72 h) leads to an increase in senescent cells as determined by SA β-Gal staining (Figure 6A) and immunofluorescence staining of PML expression (Figure 6B). A significant cell cycle arrest after LBR knockdown could not be confirmed for MEL-JUSO. There is only a tendency for a G1 cell cycle arrest in MEL-JUSO transfected with siLBR (72 h) (Figure 6C, for the percentage of cells see Figure S1B). Similar to LMNB1, the low senescence induction may also be related to compensation by EMD (Figure S2A,B). Since we could show that LBR has similar functional effects regarding senescence in melanoma-like LMNB1, we additionally investigated the expression of the LBR on the mRNA level in the senescence models described above [23,38,39]. We observed LBR downregulation in the NHEM/BRAF^V600E^ senescence model as well as in etoposide-treated MEL-JUSO cells (Figure 6D). As expected, the BRAF^V600E^-transduced, senescent NHEMs showed a closer chromatin state compared to the mock-transduced cells (Figure 6E). Consequently, chromatin state after long-term transfection (LTT) with siLBR in MEL-JUSO was analysed, and a more closed chromatin state for long-term siLBR-transfected melanoma cells could be detected compared to 72 h siLBR (Figure 6E). According to the fact that the chromatin state of BRAF^V600E^-transduced NHEMs and of the long-term-transfected melanoma cells is comparable, it could be assumed that the LTT of siLBR in melanoma has nearly similar effects compared to siLMNB1 LTT. SA β-Gal staining confirmed a significant increase in senescent cells due to siLBR LTT compared to 72 h transfection (Figure 6F). Additionally, the ratio of the percentage of SA-β-Gal-positive cells for siLBR long-term-transfected cells is also significantly increased compared to the cells transfected for 72 h (Figure 6G). As proof of concept, the increased amount of heterochromatin foci in long-term-transfected melanoma cells further supports the functional effect of LBR knockdown leading to senescence (Figure 6H). To sum up, the downregulation of LMNB1 and the LBR in MM cells leads to a significant increase in senescent cells and an alteration of the chromatin state. Accordingly, both molecules were shown to be functionally relevant in melanoma.

### 3.7. Predicted Role of the LBR in Melanoma Based on Transcriptome Analyses

**Figure 7:**
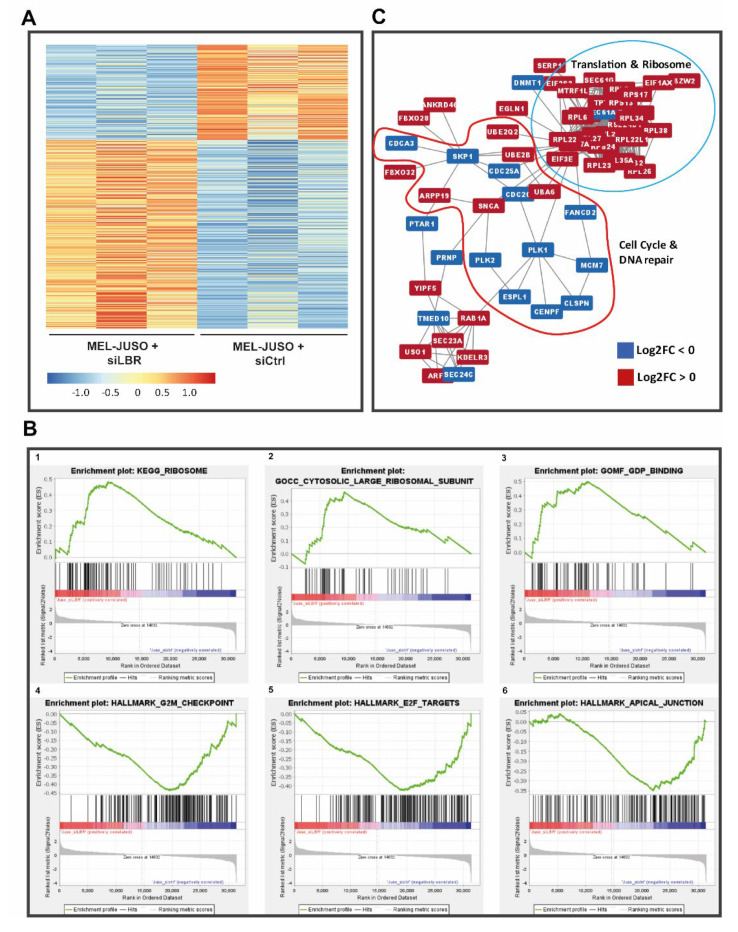
(**A**) Heatmap of differentially expressed (adj. *p* < 0.1) genes upon siLBR. (**B**) Enrichment plots (FDR < 25%) illustrating the profile of the running enrichment score (green) and positions of the enriched gene sets and the rank-ordered list of the genes differentially expressed in MM cells treated with siLBR and siCtrl, respectively, identified by GSEA. Genes upregulated in siLBR MM cells are shown on the left side of the graph in red, the downregulated ones—on the right side in blue. (**C**) Biggest cluster of the found PPIs using the list of differentially expressed genes of siLBR versus siCtrl (red: log2FC > 0; blue: log2FC < 0). The enriched functions of the clustered proteins are mainly associated with translation, protein localisation, ribosome formation and cell cycle control.

To get more information about the molecular and functional role of the LBR in malignant melanoma, we performed RNA-Seq (PRJNA841450) of the siLBR-transfected MEL-JUSO cells and the corresponding control cells. LBR knockdown efficiency was confirmed with qRT-PCR before sequencing library preparation (Figure S1B). A differential expression analysis which showed the up- or downregulation of the genes regarding LBR knockdown compared to siCtrl was performed as described above (Figure 7A). DESeq2 analysis [29] (adj. *p* < 0.1; differentially expressed genes (DEGs) in (Table S1)) resulted in 379 upregulated and 192 downregulated genes which are illustrated in a heatmap depicting the log2-fold changes of all significant differentially expressed genes (adj. *p* < 0.1, log2-fold change > 0 [red] or < 0 [blue]) (Figure 7A). Additionally, the resulting target gene expression of MMP16 and LIFR were also validated with qRT-PCR in siLBR-transfected MEL-JUSO (Table S2). Next, we assigned biological meaning to the identified differentially expressed genes by GSEA [30,31] and detected 19 enriched gene sets, eight in siLBR-transfected cells and 11 in the siCtrl-transfected ones (Table S2). The enriched gene sets in LBR-knockdown samples are mainly related to ribosome structure and function (Figure 7B 1–3), e.g., expression of different RPLs (large ribosomal subunits) (Figure 7C) is induced after siLBR transfection. This indicates the involvement of the LBR in protein synthesis. In contrast, gene sets enriched in siCtrl and thus downregulated in siLBR transfected cells show, in particular, a participation in mitotic and cell cycle processes like G2M checkpoints, E2F targets and apical junction. These mechanisms seem to be reduced in their activity in siLBR-transfected cells (Figure 7B4–6). The differentially expressed genes of siLBR versus siCtrl result in a multifarious PPI map of the corresponding gene products found by STRING application [32] (Figure 7C). A total of 721 PPIs was found with no obvious PPI clusters or subnetworks. Therefore, the PPI map was clustered via the MCL cluster algorithm of the STRING application (granularity: 4) resulting in 37 PPI networks with more than two members. Clusters of 18, 13, nine (2×), eight and seven (2×) members are enriched with the STRING application for integrin and ECM interaction, focal adhesion and catenin beta 1, kinesin-like proteins for the function of the mitotic spindle, Wnt signalling (contains the LBR), DNA repair and effects on the cell cycle, actin binding and polymerisation and stress fibres, respectively. The biggest cluster of PPIs with 62 members seems to be relevant again for translation and ribosome formation. Thereby, a tight PPI connection to biological processes relevant for cell cycle progression and control is obvious.

## 4. Discussion

LMNB1 and the LBR are proteins of the nuclear envelope and involved in important molecular processes like cell cycle regulation, cell differentiation, functional genome organisation, gene expression and DNA repair [13,41,42,43,44,45]. LMNB1, as well as LMNA/C and B2, are classified as nuclear lamina proteins, whereas the LBR belongs to the integral membrane proteins [16]. The role of LMNA is well studied in melanoma [46,47], however the role of LMNB1 and the corresponding receptor LBR remained unclear so far. In this study, we revealed that LMNB1 and the LBR are higher expressed in melanoma compared to normal human epidermal melanocytes (NHEMs). Therefore, we hypothesize that melanoma cells need a higher expression of these molecules for generating or/and keeping tumour features. The connection between LMNB1 and LBR upregulation in melanoma and changes in chromatin organisation or stability described in this study were not shown before. In short-term and long-term siRNA transfection experiments against LMNB1 and the LBR, the long-term experiments resulted in even stronger effects in our functional assays. The general rather low senescence induction may be associated with compensation of molecular tasks by EMD, which is also a nuclear structure protein. EMD is a family member of lamina associated proteins like the LBR and has nearly similar function [48]. Interestingly, EMD bind to lamins at the inner nuclear membrane and regulates gene expression by regulating chromatin architecture [49]. Followingly, EMD tend to be upregulated on protein level in 72 h siLMNB1 or siLBR transfected SK-MEL-28 which supports our hypothesis (Figure S2A,B). It became clear by our results that changes in the chromatin structure like the development of heterochromatin foci or influence on chromatin condensation are dependent on LMNB1 and the LBR in melanoma and a constant downregulation by long term treatment leads to structural and functional changes. The determined changes in heterochromatin structure are underlined by the fact that we determined a significantly increased amount of histone H3 after LMNB1 and LBR knockdown. The upregulation of H3, one of the chromatin building blocks, could further be connected to epigenetic regulation. Interestingly, acutely hampered chromatin is described to cause senescence and being involved in age-related diseases including cancer [3]. The histone H3 is prone to posttranslational modifications, leading to H3 activation [50,51]. Here, the LBR was shown to interact with H3 methylated at lysine 9 (H3K9) [52,53]. The LBR was also reported to be mislocalized and downregulated in senescence induced cervical cancer cells by the protease inhibitor MG132 and therefore might play a key role in cellular senescence [54]. In agreement with our data, LMNB1 is discussed as a potential biomarker for detecting senescent cells due to its loss in human and murine cells undergoing senescence after DNA damage, replicative exhaustion or oncogene expression [55,56]. Additionally, cells in senescence are reported to possess predominantly heterochromatin, the so called senescent-associated heterochromatin foci (SAHF), which we also determined [57]. Our data are further in accordance to previously published manuscripts as both molecules, the LBR and LMNB1, are mentioned in association with cellular senescence and ageing [13,58] in different cancer types like PC [17], breast cancer [16], HCC [18] and adenocarcinoma [59]. As we already pointed out, our long-term knockdown experiments showed stronger effects than the short-term siRNA experiments. This is confirmed by the established long-term senescence model using Mock- and BRAF^V600E^ lentiviral-transduced NHEMs. Therefore, we hypothesize that a robust long-lasting LMNB1 or LBR downregulation is necessary to create a strong chromatin change and consequently a senescence phenotype in melanoma. Conclusively, melanoma cells express high levels of LMNB1 and LBR to prevent senescence. Additionally, we found LMNB1 and LBR to be downregulated in the melanocytic BRAF^V600E^ senescence model as well as in etoposide-induced senescence model in melanoma cells. LMNB1 is discussed as a senescence marker in general whereas the role of LBR as a senescence marker was first shown by this study. Thus, we newly elucidated the role of both genes as potential senescence markers in melanoma. Similar effects were observed by Lukášová et al., showing that LMNB1 expression is reduced in fibroblasts after senescence is induced by γ-irradiation [13,15]. In summary, we could not only show that knockdown of LMNB1 or LBR in melanoma cells leads to senescence but also vice versa, LMNB1 and LBR are significantly downregulated in our senescent models supporting a function in regulation of senescence. 

The analysis of differentially expressed genes in siLMNB1 transfected melanoma cells compared to siCtrl was performed to understand these processes in molecular detail. Some of the defined target genes of LMNB1 like LIFR and IL6R are already reported to be cancer associated [60,61]. LIFR belongs to the type I cytokine receptor family and could form a receptor complex which is involved in important cellular processes like differentiation, proliferation, and survival [62]. The LIF/LIFR axis is reported to be involved in the maintenance of stem cells [62]. Maybe the loss of LIFR due to LMNB1 knockdown results in reduction of the stemness like properties of melanoma cells leading to senescence. In fact, the significant downregulation of LIFR according to LMNB1 knockdown underpins the loss. This assumption is further supported by a study of Kuphal et al., where LIF is shown to be moderately expressed in melanoma cell lines of primary and metastatic origin and in melanoma tissue [63]. Therefore, a loss of LIFR might hamper the LIF/LIFR axis. We also observed upregulated genes in siLMNB1 melanoma cells, like MYL12B. According to Dabrowska et al., MYL12B upregulation could be linked to senescent colon cancer cells [64]. Consequently, the upregulation of MYL12B might also be involved in the functional effects due to LMNB1 knockdown. Moreover, based on our GSEA results, LMNB1 expression plays a role in translational processes, ribosomal activity, and protein targeting. The subsequent STRING analysis for PPI determines LMNB1 to be relevant for translation and ribosome formation as well. Interestingly, disregulation of ribosome biogenesis could be associated with senescence and cell cycle arrest which suits the bioinformatic results [65]. LMNB1 downregulation might lead to a significant enrichment of several genes involved in these biological processes and corresponding pathways. Translation and ribosomal protein synthesis are important cellular processes, which depend on each other. It is known that there are oncogenic pathways which influence translation and metabolism like c-Myc, RAS and PI3K-mTOR by glycolysis and protein synthesis [66]. The upregulation of genes, which are involved in these processes due to LMNB1 knockdown, could influence the interplay between translation and metabolism leading to changes in oncogenic pathway regulation. 

The analysis of differential expression pattern in LBR-knockdown cells show 379 upregulated and 192 downregulated target genes compared to siCtrl, respectively. Several differentially expressed genes are involved in ribosomal organisation and translation like the ribosomal proteins (RPLs). Therefore, LBR also plays a role in translational processes within the cell. Interestingly, RPL5 is determined to act tumor suppressive in various cancer types, also melanoma [67] and RPL34 seems to be tumour suppressive in esophageal cancer [68]. The predicted upregulation of RPLs due to LBR knockdown indicates a tumor suppressive release regarding senescence and reduced proliferation. Therefore, it could be assumed that LBR knockdown is involved in stress of the endoplasmic reticulum (ER stress). Interestingly, a recent study revealed that activation of ER stress pathway mediates a resistance mechanism in uveal melanoma [69]. ER stress was also determined to be involved in induction of senescence or cell death in osteoarthritis chondrocytes or osteoarthritic cartilage [70,71]. Therefore, LBR knockdown could eventually be related to ER stress activation, leading to senescence. The hypothesis that LBR expression might play a role in processes regarding the ER and translation, is further supported by the results of GSEA revealing the upregulation of gene sets involved in KEGG ribosome pathway. Interestingly, E2F targets and G2M checkpoints are predicted to be downregulated in siLBR transfected melanoma cells. E2F includes seven transcription factors which are known to play an important role in cell cycle progression and cancer [72,73]. Moreover, in mammalian cells, the E2F factors serve as strong regulators of cell-cycle checkpoints and its deregulation leads to a loss of checkpoint controls which is a hallmark of cancer [74,75]. Consequently, the predicted downregulation of E2F targets in LBR-knockdown cells might influence the E2F activity which leads to disruption of cell cycle checkpoints. Therefore, it could be assumed that the G1-S transition and G2-M transition are influenced by LBR knockdown leading to mitotic catastrophe and DNA damage which could be associated to our functional determinations. The determined PPI with STRING application could also be linked to biological processes relevant for cell cycle progression and control. Summing it up, LBR downregulation in melanoma might cause an upregulation of several genes involved in translational processes, resulting in ER stress and affecting mitotic processes, eventually leading to the functional results we determined.

## 5. Conclusions

In this study, we could show that melanoma needs LMNB1 and the LBR for translational processes. Our analysis newly revealed that a downregulation of LMNB1 and the LBR might regulate mitotic processes and based on defects in replication, lead to cellular senescence and changes in chromatin state in malignant melanoma. LMNB1 and the LBR are functionally relevant for melanoma cells to prevent them from cell cycle arrest through influencing the regulation of several genes. Summing it up, both molecules indirectly influence different important pathways leading to similar functional effects, although partly affecting different target genes. 

## Data Availability

I: RNA sequencing data will be deposited in the SRA Submission Portal database prior to publication.

## Supplementary Materials

The following supporting information can be downloaded at: https://www.mdpi.com/article/10.3390/cells11142154/s1.
