# Supplementary material for: Knockdown of Lamin B1 and the Corresponding Lamin B Receptor Leads to Changes in Heterochromatin State and Senescence Induction in Malignant Melanoma
Source: Cells. 2022 Jul 8;11(14):2154. doi: 10.3390/cells11142154 (PMC9321645; doi:10.3390/cells11142154)
Supplement: Supplementary file 1 [file cells-11-02154-s001.zip › Supplementary figure S1.pdf]

A

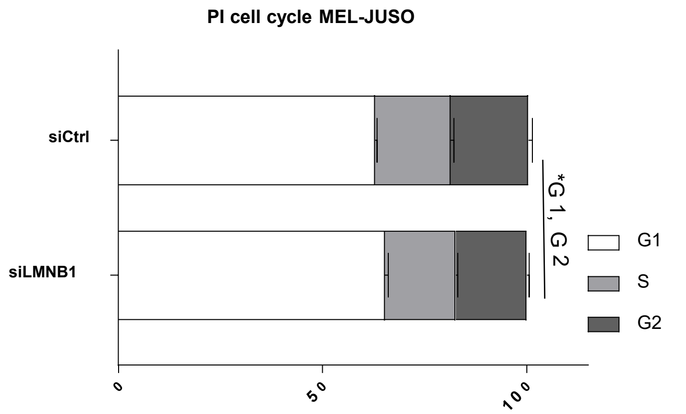

MEL-JUSO		G1 %	S/G2 %
siCtrl	n=1	63.774115	36.225885
	n=2	62.03925	37.96075
	n=3	62.907175	38.61691
siLMNB1	n=1	66.759865	33.24013
	n=2	63.682495	36.317505
	n=3	65.39983	34.60017

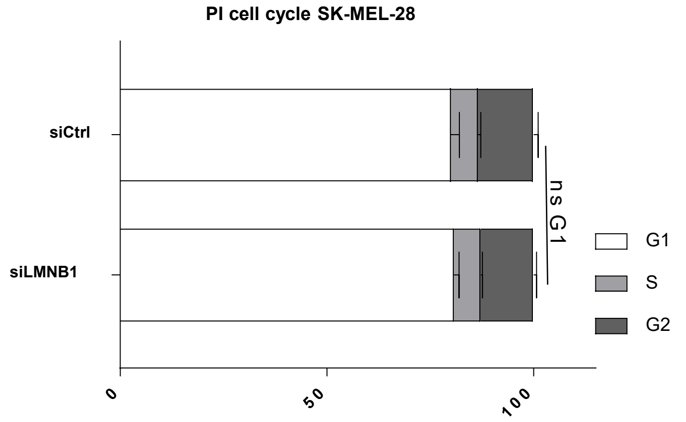

SK-MEL-28		G1 %	S/G2 %
siCtrl	n=1	84.0262916	15.9737085
	n=2	78.6462662	21.3537339
	n=3	77.6485098	22.3514903
siLMNB1	n=1	82.9967854	17.0032146
	n=2	79.1606754	20.8393247
	n=3	80.344609	19.6553912

B

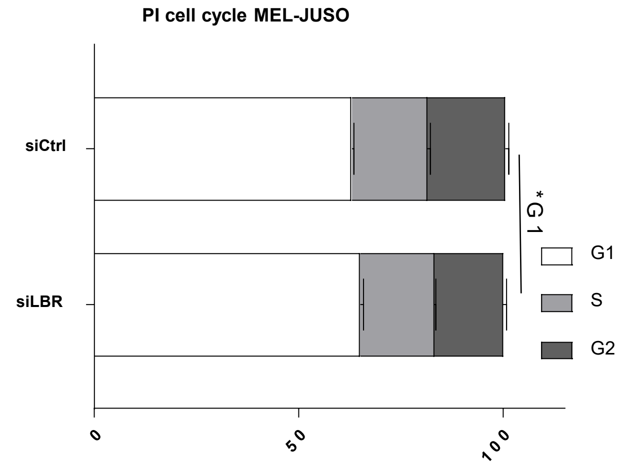

MEL-JUSO		G1 %	S/G2 %
siCtrl	n=1	63.774115	36.225885
	n=2	62.03925	37.96075
	n=3	62.907175	37.0928272
siLBR	n=1	66.2519	33.748095
	n=2	65.14242	34.857585
	n=3	63.80766	36.19234

Figure S1: (A) Flow Cytometry with the fluorescent dye propidium iodide for cell cycle staining. MEL-JUSO and SK-MEL-28 treated with siLMNB1 and siCtrl for 72 h. Table show the percentage of cells in cell cycle phase respectively. (**B)** Flow Cytometry with the fluorescent dye propidium iodide for cell cycle staining. MEL-JUSO treated with siLBR and siCtrl for 72 h. Table show the percentage of cells in cell cycle phase respectively. (2way ANOVA and subsequent Uncorrected Fisher's LSD Test). Bars represent mean±SEM (*= p≤ 0.05 and ns = not significant))
