# Supplementary material for: Knockdown of Lamin B1 and the Corresponding Lamin B Receptor Leads to Changes in Heterochromatin State and Senescence Induction in Malignant Melanoma
Source: Cells. 2022 Jul 8;11(14):2154. doi: 10.3390/cells11142154 (PMC9321645; doi:10.3390/cells11142154)
Supplement: Supplementary file 1 [file cells-11-02154-s001.zip › Supplementary figure S2.pdf]

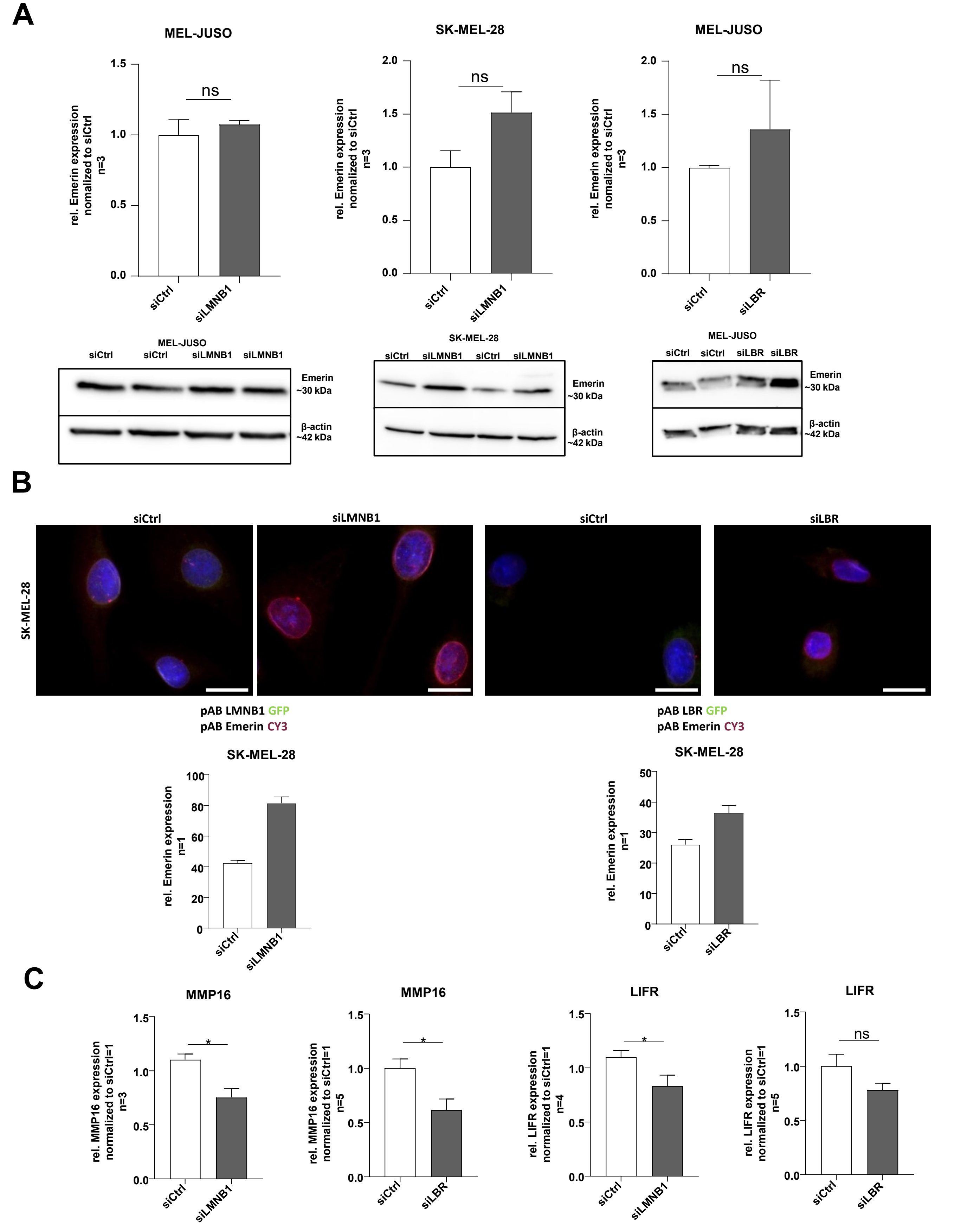

Figure S2: (**A**) Western Blot analysis of Emerin protein expression in melanoma cell lines MEL-JUSO and/or SK-MEL-28 treated with siLMNB1 and siLBR for 72 h (Student's t-test, Bars represent mean+SEM). (**B**) Immunofluorescent staining of Emerin expression (CY3) in SK-MEL-28 transfected with siLMNB1 (GFP) or siLBR (GFP) and siCtrl for 72 h (n=1, Bars represent Bars represent mean ± SEM within the biological replicate). (**C**) Quantitative real-time PCR analysis of target genes expression due to LBR knockdown for 72 h in MEL-JUSO and SK-MEL-28 treated with siLMNB1 and siLBR for 72h (Student's t-test, Bars represent mean+SEM). (*= p≤ 0.05 and ns = not significant).
