# Supplementary material for: Knockdown of Lamin B1 and the Corresponding Lamin B Receptor Leads to Changes in Heterochromatin State and Senescence Induction in Malignant Melanoma
Source: Cells. 2022 Jul 8;11(14):2154. doi: 10.3390/cells11142154 (PMC9321645; doi:10.3390/cells11142154)
Supplement: Supplementary file 1 [file cells-11-02154-s001.zip › Supplementary figure S3.pdf]

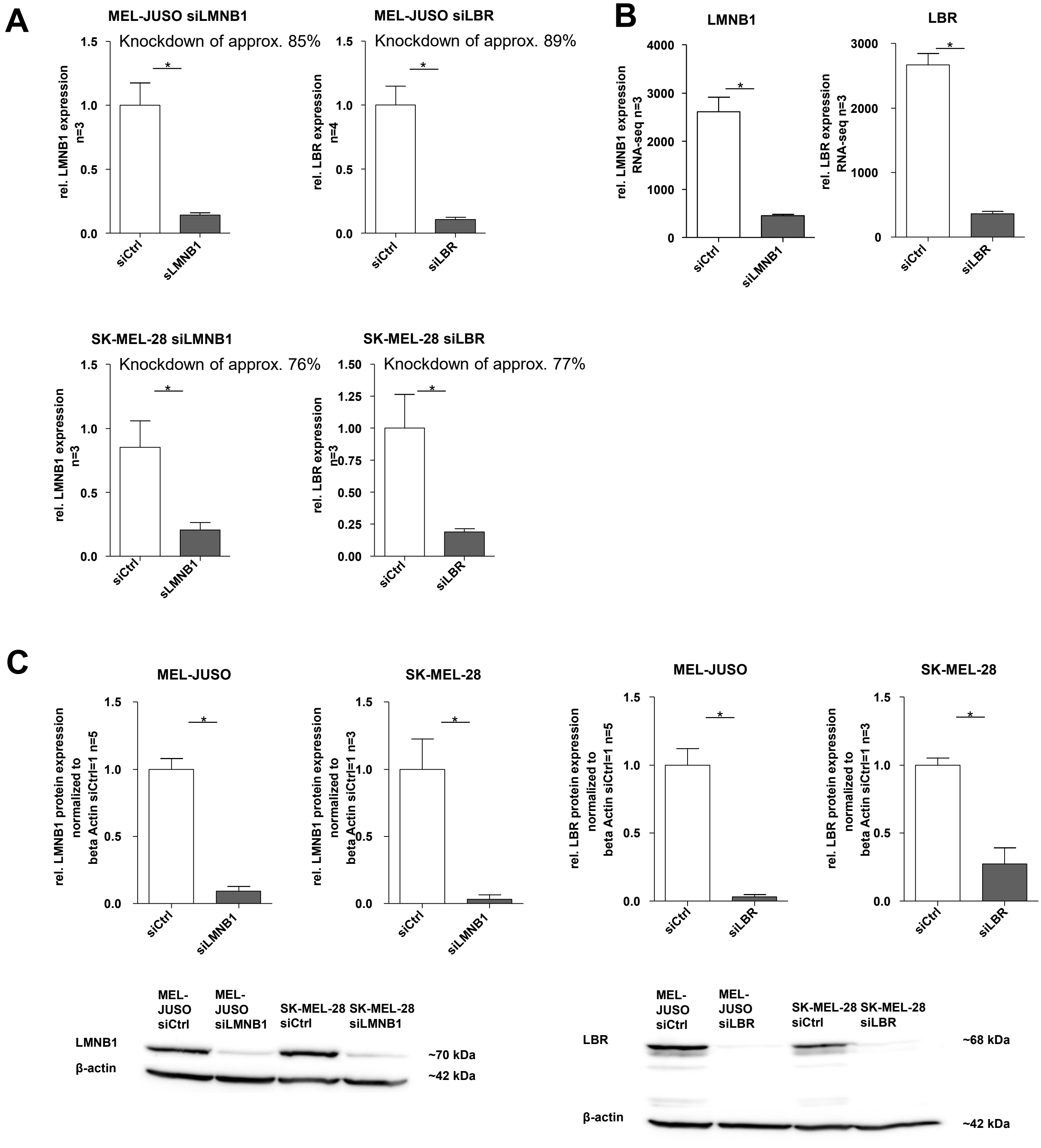

Figure S3: (**A**) Quantitative real-time PCR analysis of LMNB1 and LBR RNA expression in MEL-JUSO and SK-MEL-28 treated with siLMNB1 and siLBR for 72 h. Bars represent mean+SEM, (statistical significance was calculated using t-test with ΔCP values indicated as *= p≤ 0.05). (**B**) RNA-sequencing analysis in primary melanoma cells (MEL-JUSO) treated with siCtrl, siLMNB1 or siLBR. LMNB1 and LBR expression compared to siCtrl for knockdown verification. Bars represent mean+SEM, (statistical significance was calculated using t-Test with ΔCP values indicated as *= p≤ 0.05). **(C**) Western Blot analysis of LMNB1 and LBR protein expression in MEL-JUSO and SK-MEL-28 treated with siLMNB1 and siLBR for 72 h. Bars represent mean+SEM, (statistical significance was calculated using t-test with ΔCP values indicated as *= p≤ 0.05).
